# Do expert surgeons use mental skills to improve their surgical performance?

**DOI:** 10.1007/s44186-025-00392-4

**Published:** 2025-09-27

**Authors:** Nicholas E. Anton, Ashley M. Yurco, Kareem A. Kashif, Brisa Urquieta de Hernandez, Charles Brown, Dimitrios Stefanidis

**Affiliations:** 1 Department of Surgery at the Indiana University School of Medicine, 545 Barnhill Dr., Indianapolis, IN 46202 USA; 2 Medical Intensive Care Unit, Atrium Health, Charlotte, NC USA; 3 Mount Sinai Medical Center, Miami, FL USA; 4 Lloyd H. Dean Institute for Humankindness and Health Justice, CommonSpirit Health, Chicago, IL USA; 5 Get Your Head In The Game Inc, Charlotte, NC USA

**Keywords:** Surgery, Expert surgeon, Mental skills, Stress management, Performance enhancement

## Abstract

**Purpose:**

Surgeons often experience stress that may negatively impact performance. Mental skills are psychological techniques designed to prevent skill deterioration under stress and enhance performance. Mental skills curricula have proved effective in other disciplines but are rarely used in surgery. Additionally, research on mental skill use by surgeons has been very limited. The objectives of this study were to (1) explore whether expert surgeons use mental skills to enhance their performance, (2) determine how they implement mental skills, and 3) assess the need for mental skills training in surgical residency.

**Methods:**

After IRB approval, 7 expert surgeons (≥ 15 years of experience, prominence in their respective fields) from general surgery, obstetrics/gynecology, and neurosurgery voluntarily participated in semi-structured interviews. Responses were transcribed and analyzed until themes were identified.

**Results:**

All interviewed surgeons indicated that they regularly use some combination of mental skills to achieve their ideal performance state for surgery, manage intraoperative stress, and manage distractions before and during surgery. Further, all participating surgeons reported feeling responsible, as the leader of the surgical team, to project a calm demeanor during stressful situations to optimize the team’s performance. While none of the participating surgeons had received mental skills training, 71% (5 of 7) advocated for the incorporation of mental skills training into surgery residency.

**Conclusions:**

Mental skills are routinely used by expert surgeons to enhance performance and manage stress. While mental skills seem to be acquired over years of practice, experts agree that these skills warrant formal introduction during residency.

## Introduction

Stress, the cognitive perception of a situation as threatening or distressing and a lack of ability to cope with the situation, has received increasing attention in the surgical literature as a significant barrier to successful surgical performance [1]. Due to the myriad of inherent challenges of surgery, surgeons are susceptible to experience heightened stress, which can negatively impact fine-motor dexterity, clinical decision making, leadership and communication skills, and concentration among other factors, which can lead to technical errors that compromise patient safety [2–4]. One potential theoretical explanation for how stress can impact cognitive processes related to surgical performance (e.g., clinical decision making, situation awareness, leadership, etc.) is based on cognitive load theory [5].

Heightened stress has been shown to increase cognitive load and tax working memory capacity, or the amount of information that an individual can shift attention between at any given time [6]. Overwhelming stress can lead to cognitive overload, which can impair working memory and lead to attentional narrowing (i.e., “tunnel vision”), decreased sensitivity for relevant peripheral information, and hypervigilant decision-making, which is characterized by impulsive, disorganized thought processes [5, 7]. A previous study on surgeons’ experiences with intraoperative stress in the operating room found that 40% of respondents had witnessed an intraoperative error caused by stress [8]. Thus, helping surgeons develop strategies to manage stress and the associated cognitive overload may have important implications for patient safety.

Mental skills are psychological tools and strategies intended to help performers consistently achieve their optimal mental state for performance, leading to the reliable execution of skills regardless of the situation [9]. In the context of surgical performance, mental skills are distinguishable from cognitive skills. While cognitive skills refer to constructs such as situation awareness or decision making, mental skills are designed to help performers reduce stress and enhance performance, and consist of specific techniques such as goal setting, relaxation, attention management, mental imagery, refocusing techniques, and performance routines, among others [10, 11]. Relaxation techniques include strategies like diaphragmatic breathing to reduce the physiological effects of acute stress [12]. Attention management techniques (e.g., reframing negative or maladaptive thoughts to more positive and task-relevant thoughts) maintain rational optimism for performance and redirect attention to stepwise sequences of actions involved in an action plan. Finally, mental imagery refers to the rehearsal of techniques in the mind in the absence of physical stimuli that enables pre-performance planning and the development of confidence.

Mental skills training programs have been implemented efficaciously with high-performers in various high-stakes domains, including the military [13, 14], police special forces [15], and elite athletics [16]. In recent years, mental skills and emotional regulation training programs have been implemented with surgical trainees, and have been shown to effectively enhance attention, reduce stress, and optimize performance during stressful situations [17–21]. However, despite the body of validity evidence showing the benefit of dedicated psychological skills education for trainees, little is known about whether practicing surgeons use mental skills to enhance their performance and manage stress and with what frequency. It is possible that expert surgeons have learned to use these skills serendipitously and use them subconsciously.

Expertise in surgery is characterized by a mastery of extensive and dynamic knowledge, skills (e.g., technical, cognitive, and interpersonal), and competencies [22], which are developed through a combination of substantial experience, innate ability or aptitude for the aforementioned factors, and deliberate practice to optimize the execution of these factors in on-demand performance situations [23]. Thus, the ability of expert surgeons to consistently execute these skills at an elite level under challenging clinical situations suggests that they have likely developed effective mental skills as a result of their experiences. Determining how expert surgeons utilize mental skills to optimize surgical performance is critical, as it may help identify techniques that warrant inclusion in surgical training to optimize trainees’ surgical performance much earlier in their careers. Unfortunately, there is a paucity of studies investigating the specific mental skills expert surgeons use to manage stress and enhance performance. The purpose of this study was to explore whether or not expert surgeons use mental skills to enhance their performance, how they implement these skills prior to and during surgery to optimize performance and obtain their opinions on the necessity of formal mental skills training during surgical residency.

## Methods

After Institutional Review Board approval, expert surgeons were approached via email and were asked to voluntarily participate in individual, semi-structured interviews. We conducted interviews either in person or via teleconference, which were video recorded to ensure accuracy during transcription and allow for direct quotation. To be considered for inclusion, surgeons had to have ≥ 15 years of surgical experience and were considered by the study team to have expertise in their respective disciplines (i.e., holding leadership positions at the international, national, or institutional levels). One study team member, a leader in surgical education at the international and institutional levels, identified prospective expert surgeons and proposed their study inclusion to other study team members. When consensus was achieved on their inclusion in the study, participants were contacted electronically to solicit their voluntary participation. Three co-investigators transcribed and cross-validated responses. All interviews were de-identified prior to transcription.

A performance psychologist with over 30 years of experience in mental skills training developed the interview questions, focusing on surgeons’ use of various mental skills. The questions were vetted by a surgeon educator and the final guide (see Appendix A) was used to direct the interviews. The guide was distributed to participants prior to the interview for their review. The interviews were scheduled at the participants’ convenience and performed in a conversational style, allowing for prompting to explore pertinent concepts further. We asked participants about their general thoughts on how mental factors, including the use of various mental skills, influence surgery and help manage stress individually and as the leader of the surgical team. Also, we asked participants to consider the potential value of mental skills training for surgical trainees.

Thematic analyses of the interviews were performed independently by three co-investigators who were supervised by a qualitative research expert. Thematic analysis, which accommodates both inductive theme emergence and deductive coding from predetermined frameworks, was appropriate given our exploratory research questions and the need to balance structured inquiry about known mental skills with openness to unexpected findings from our expert interviews [24]. The qualitative research expert educated the study team on qualitative coding of semi-structured, in-depth interviews. After interview transcriptions had been completed, the research team and the qualitative research expert independently analyzed one of the interviews. These analyses were then compared and contrasted to the qualitative expert’s review to ensure all raters were consistent in their coding scheme. A common coding scheme was developed using a priori codes derived from the interview guide, and the co-investigators reviewed and coded the remaining six interviews. At least two raters coded each interview, and weekly meetings were held during the coding process to confirm themes and ensure the trustworthiness of findings. During these meetings, common themes and direct quotations were selected to lend support to the researchers’ interpretations. Individual and team-focused mental skills were highlighted based on the frequency by which they were mentioned during interviews. In the weekly research team meetings, discussions on data saturation guided the team to determine whether additional interviews were needed. Once a consensus was achieved by the team on data saturation of key themes, the team concluded interviews. Analysis of the interviews revealed key themes that emerged across participants, which are presented below with their frequency of occurrence.

## Results

A total of seven expert surgeons, representing several specialties including general surgery (*n* = 4), obstetrics/gynecology (*n* = 2), and neurosurgery (*n* = 1), were interviewed. The average interview duration was 46 ± 9 min. Most interviewed surgeons (6 of 7) were male. Two surgeons held the chair position in their respective departments, one surgeon held medical director of trauma services at their institution, and one surgeon served as the past president of the American College of Surgeons. The three remaining surgeons held residency program or division leadership positions.

**Fig. 1:**
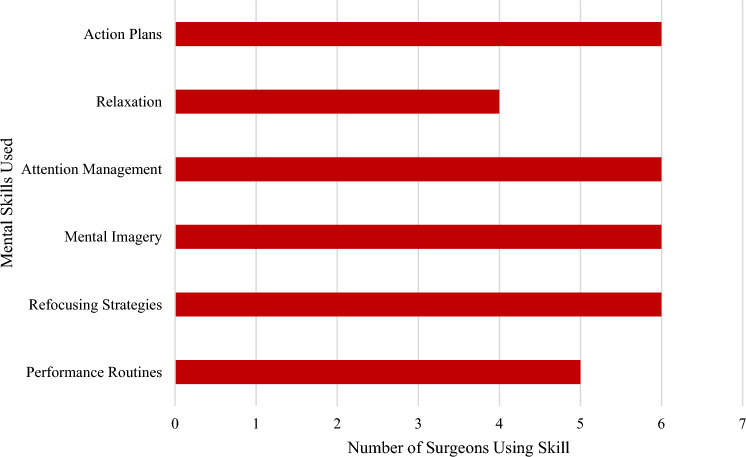
Expert Surgeons’ Use of Mental Skills

All surgeons interviewed for this study indicated they regularly use some combination of mental skills such as mental rehearsal, attention and thought management techniques, relaxation strategies, action plans, refocusing strategies and performance routines to achieve their ideal performance state for surgery, manage intraoperative stress, and manage distractions before and during surgery (see Fig. 1).

### Attention management

Attention management strategies were unanimously reported as helpful in maintaining vigilance and a balanced mind throughout surgical procedures, preventing negative self-talk, loss of confidence, and loss of focus amidst chaotic and stressful situations in the operating room. One participating surgeon described the importance of maintaining focus during all phases of an operation:

*“You have to have that attention, that focus, that voice in the back of your head that says, ‘Something could go wrong at any time with this and hopefully you have thought that through.’ … For whatever operation or for whatever phase of the operation you are in, you have got to maintain focus throughout every phase of the operation because they are all important. Things can happen badly at any phase of those operations and you need to be prepared to deal with that.”* [Participant N1V]

Similarly, another surgeon echoed this concept of controlling one’s focus to maintain performance during deteriorating conditions:

*“I think the most important thing is that you have to have balance of thought. You can’t let your mind start racing and lose concentration whether it’s during the flow of an operation… certainly in times when you have events that require you to rescue out of a problem.”* [Participant 6WF]

One skill that may enable surgeons to maintain focus on the steps of an operation or refocus in the event that concentration is lost is mental imagery.

### Mental imagery/rehearsal

Of the specific mental skills discussed, participants (six of seven) indicated they regularly use mental imagery to prepare for cases, or to refocus if they become distracted during a procedure. Regarding mental preparation, participating surgeons described how imagery can help them rehearse the technical steps of a procedure and consider possible contingencies to intraoperative complications:

*“(Mental imagery) allows you to operate without touching the patient because you’ve mentally gone through the procedure… it allows you to reduce your stress level because if you can do those things and you have the ability to run through the case and visualize the instruments you need and the suture you need and what problems you might have it is going to lower your anxiety level.”* [Participant U3J]

Mental imagery may also be an effective technique to aid surgeons’ ability to refocus their attention back to their action plans effectively, as described by one of the participants,

*“You usually take a step back, at least mentally take a step back. I wouldn’t call it a time out but take a step back and rethink the situation. All the mental rehearsal that you do, you sort of go through the cycle of events you rehearse.”* [Participant 6WF].

### Refocusing strategies

Also, six of seven surgeons reported using refocusing strategies to redirect attention back to the patient if faced with intraoperative distractions that lead to loss of focus:

*“There’s a few cases where I’ve had either arterial venous malformations or aneurisms that would rupture in the middle of a case and things would get a bit spooky and you kind of have to mentally force yourself just to ignore a certain amount of the chaos, focus, get back on track and go as if nothing really happened.”* [Participant 8CK]

When utilizing mental imagery to prepare for a surgical case or refocus attention when faced with loss of concentration, expert surgeons implement action plans to break down procedures into manageable steps.

### Action plans

Action plans, the process of identifying the stepwise actions required to execute a procedure, were discussed by all interviewed surgeons. Participants detailed how action plans allow them to think through procedures ahead of time, identify potential challenges and solutions, and ensure the entire surgical team is prepared. One participant described this process by saying,

*“You really do have to break things down into steps and think about what you are going to need for this step, how’s that going to impact what everybody else is doing, and anticipate those needs and get those needs verbalized.”* [Participant N1V].

In addition to the usefulness of mental imagery of action plans to mentally prepare for procedures, performance routines are often implemented by expert surgeons to consistently get in the right mindset for surgery.

### Performance routines

Six of seven expert surgeons described the use of performance routines to help facilitate their focus and reliably achieve their ideal mental state for performance:

*“I get very comfortable when I do the same thing over and over and over again, and that can be as simple as my routine in the morning before surgery… make sure I have a pattern that I follow because that pattern helps me to focus more when I get to the next step.”* [Participant 6WF]

### Relaxation skills

The last mental skill reported was relaxation techniques, which was used by five of seven expert surgeons to reduce the psychophysiological response to stress. One surgeon indicated that.

*“I think that, for me personally, the intensity climbs knowing what the surgery is leading into or what the next stage is, … and then I have to do something to bring it down.”*

When the same surgeon was asked if any specific skills help in handling stress, he replied, *“Probably nothing more dramatic than taking a couple of deep breaths or a sigh. That seems to, especially under the microscope, that allows me to kind of pause, catch up a little bit, and then settle in.”* [Participant 8CK].

### The scrub sink

In addition to the above-mentioned mental skills, several emergent concepts were identified in reference to how participants use these skills. The scrub sink was often identified (six of seven) as a place to relax, mentally prepare for upcoming cases, and block out distractions:

*“As we approach the operating room, and we are dealing with the floor calling, with this question and that question, all this chaos, your beeper, your cellphone, this phone and that phone, again, I go to the scrub sink and - I’ve never thought of it this way before - but I wash all that stuff away and I take a cleansing breath.”* [Participant W2F]

Another participant described a similar experience of eliminating the stressors and distractions while mentally preparing for a case by explaining,

*“What I find is as I am scrubbing or as I am getting ready for each procedure, I am starting to think about what I am going to do and I am mentally going through steps… as I do that, these other things (distractions) just fade away.”* [Participant U3J].

One participant explained that the scrub sink is an ideal place to mentally prepare for surgery:

*“You’re doing a very mundane task, you’re just sitting there washing your hands and the fact that you can take that time to do something with it is very helpful. And if you can begin to mentally prepare the steps of the surgery in your mind and then what you’re going to encounter along the procedure, I think it just helps the whole process play out more routinely and easier once you get it.”* [Participant 8CK]

Individual mental skills, while effective in optimizing cognitive performance and reducing stress during performance, require self- and situation awareness to recognize when it is necessary to implement these skills.

### Situation awareness

All participants independently identified awareness as a critical cognitive factor for surgical performance. One level of awareness that emerged repeatedly was the surgeons’ self-awareness over their cognitive and emotional state and the awareness to recognize the need to utilize mental skills to relax and maintain cognitive balance:

*“Once you have that awareness that you’re getting tense and things are going the wrong direction, that’s the time when you can kind of direct it a little bit and say, ‘Okay, let’s take a couple of deep breaths and pause here a second and then start back into it with a little more even keel’.”* [Participant 8CK]

Self-awareness also seems to interact with situation awareness and awareness of the team in the form of empathy:

*“People have to have awareness of their environment, they have to have self-awareness, some of these other skills that go towards displaying confidence, don’t have anything to do with how well you scored on a test, but how well you understand the environment that you’re in and that people’s emotions as they plan the situation. The traditional surgical environment was that you had a surgeon that came in screaming and yelling at everybody, and got everything he wanted and that everybody was on edge and they did their thing, but that’s not professional or the right work environment and so part of this is fostering the group as a whole.”*[Participant 6WF]

Situation awareness and awareness of the team's emotional state may be important factors for expert surgeons to consider when it is necessary to intervene and project a calm demeanor to reduce the team's arousal level.

### Leadership/Team-based skills

All participating surgeons felt responsible, as the leader of the surgical team, to project a calm and controlled demeanor during stressful clinical situations to help the team perform their best. Individual mental skills may aid surgeons in their ability to lead the surgical team effectively.

*“I think from an emotional standpoint, it is very easy for us to get caught up in all of the implications of what is going on, but I also do believe that the team takes its cues from the team leader, and that is elective or emergent, and so if I communicate, whether intentionally or unintentionally, by my behavior, by my words, by my actions, if I communicate insecurity, nerves, not being sure of what, not being in control of the situation, easily gets transmitted to the rest of the team.”*[Participant N1V]

Similarly, one participant expressed that it was imperative for a surgeon to project a calm demeanor to lead the team effectively during stressful situations:

*“I think it’s very important that whoever assumes leadership (and is) in charge in a situation like this, … shows calmness. Because it infuses calmness in the rest of the team immediately… I am not calm, I’m scared, I’m nervous, all those other feelings are inside of me, but I’m telling myself, ‘Look, you’ve been there before and did fine. These situations tend to resolve themselves. Remain calm.’ By saying that to me, I calm myself so my voice doesn’t project as a voice that is not calm. So they then recover, but I tell them, ‘We have a problem and I need everybody’s attention.’ Then everybody is quiet.”*[Participant E3Z]

### Value of mental skills training

Importantly, while none of the participating surgeons had received formal mental skills training, five of seven advocated for the incorporation of mental skills training into surgery residency curricula. One expert surgeon explained, *“This (mental skills training) isn’t taught. I think this is a huge deficiency within the surgical community because there are certainly lots of people who I think would benefit from it.”*[Participant 6WF] Another participant also expressed the potential value of mental skills training during residency by saying, *“I think that everyone should have mental skills training… I think that self-awareness and some exercises that weren’t in traditional curricula just anything that brings you self-awareness is really a valuable thing.”* [Participant W2F].

Interestingly, one participating surgeon did not report intentionally using any of the mental skills identified in the interview guide to reduce their stress or enhance their performance. When this surgeon was asked what they believed the mental, psychological, and emotional factors that influence successful surgery are, they responded:

*“Psychological factors, I’m not sure I can think of any. When a surgeon is doing an operation, it’s typically something they’ve done many times… I don’t know if there’s a lot of musing about it or self-introspection about how I am doing in this operation… It’s a job, you’re doing something you’ve been trained how to do and you just execute.”*[Particiant 2XW]

However, when asked how their performance strategies adapt intraoperatively, this surgeon fascinatingly responded, *“You react differently to those unplanned events. The ones that are life threatening, you have to very quickly get control, calm down, sometimes you just have to put your finger on something and call for help.”* Furthermore, when discussing the importance of remaining calm as the primary surgeon, the participant said:

*“Some of the best surgeons I have ever known in my life, never raised their voice, never got angry, never lost their cool, always in control. People that scream and yell usually aren’t always that good… If you really watch some of the best surgeons around, they’re generally pretty calm and in control.”*

This surgeon later responded that*, “If you as the surgeon lose control, the team is going to fall apart.”* Also, this surgeon described a seemingly subconscious process of focusing and blocking out distractions at the scrub sink:

*“It seems that once I start washing my hands at the sink, that’s when the switch goes off. Maybe you’ve trained yourself to, at that point the switch goes off and it’s all about the operation. I’ve never thought about it to be quite honest until you asked me, but I guess for me that’s the time to stop joking and get down to business.”*

## Discussion

The purpose of this study was to determine if expert surgeons use mental skills to enhance their performance, how they implement these skills pre- and intra-operatively to optimize performance, and obtain their opinions on the necessity of formal mental skills training during surgical residency. Our findings indicate that all interviewed surgeons routinely use several mental skills preoperatively and intraoperatively to achieve an optimal mental state for performance and manage intraoperative stress and distractions. There was one participant who initially denied using mental skills, but later described the routine use of attention management techniques to block out distractions and “flip the switch” to mentally prepare when scrubbing into a case, developing action plans to prepare for cases, and relaxation skills to calm down and maintain control during challenging clinical situations. This may indicate an unfamiliarity with the terminology being used in the field, in addition to the implicit or subconscious use of these skills in practice.

The working memory capacity literature suggests that heightened stress and subsequently, cognitive overload, can lead to attentional narrowing and decreased sensitivity to relevant information [5, 7]. The findings from this study suggest that expert surgeons use mental skills preoperatively to mentally prepare for their cases (e.g., creating action plans, engaging in mental imagery, and utilizing performance routines to reduce variability in lead up to performance) and intraoperatively to prevent attentional disruptions (e.g., attention management), to manage heightened physiological arousal (e.g., relaxation skills), and to refocus if attention shifts to task-irrelevant stimuli (e.g., refocusing strategies).

This emerging theme is congruent with the surgical literature, as researchers have found that expert surgeons are able to effectively transition from automatic cognitive processes to effortful, thoughtful performance by slowing down when faced with clinical challenges [25]. The authors explain that by slowing down during a procedure and becoming more attentive, expert surgeons are able to focus more effectively and refocus if faced with distractions during difficult situations, which enables them to maintain or regain control as the situation demands. This process is related to working memory capacity, as slowing down and focusing more attentively may coincide with the need to recruit additional cognitive resources to manage the demands of a difficult intraoperative situation. Interestingly, some participating surgeons in this study expressed a very similar sentiment. One surgeon described this process by indicating they engage in deep breathing when facing stressful intraoperative situations, which enables them to slow down and maintain effective decision-making instead of relying on reflex actions, which can exacerbate issues. Once experts recognize their stress level is rising, their use of mental skills may enable them to remain calm, slow down, and shift out of automaticity to remain in control during challenging situations, which prevents them from experiencing cognitive overload. Ultimately, preventing cognitive overload may enable surgeons to maintain their working memory capacity, cognitive balance, and attentional flexibility to attend to relevant environmental stimuli, preserve situation awareness, and maintain effective decision-making, which are all critical factors to their individual performance as well as to their ability to lead the surgical team effectively. Indeed, a theme that emerged in all interviews without direct prompts, even during the interview with the one surgeon who reported not actively utilizing mental skills, was that expert surgeons feel situation awareness is critical to effective performance of the surgical team. By maintaining situation awareness and working memory capacity, experts can recognize when it is necessary to lead the surgical team more effectively by projecting a calm and controlled demeanor.

Again, all interviewed surgeons identified that as the leader of the surgical team, they were responsible for projecting a calm demeanor during challenging clinical situations to maintain the team’s performance. This finding is salient when considering how surgical trainees learn to lead surgical teams during stressful situations, as trainees may develop maladaptive leadership techniques that persist into their practice. One surgeon expressed regret in past experiences managing residents who had committed errors, and felt this was a consequence of lacking mental skills training from mentors during their residency training.

It is possible that the inclusion of formal mental skills training in surgical education can ultimately enhance trainees’ ability to manage their own stress more effectively. Initial evidence on mental skills training with surgical novices and trainees is attestation of the benefit of dedicated mental skills curricula on participants’ ability to manage stress and maintain performance effectively [17–21]. These skills enable surgeons to maintain situation awareness of the emotional state and performance of the team, and optimally lead surgical teams during adverse events in the operating room, which has direct implications for patient safety.

Furthermore, the majority of expert surgeons interviewed in this study (five of seven) felt that dedicated mental skills training should be integrated into surgical residency training. Given the engagement of these experts in surgical education at the institutional and national levels, their opinions carry significant weight and reflect a growing desire to equip learners with methods to manage the cognitive demands of surgery early on during training. Accordingly, stakeholders in surgical education should consider adopting a mental skills training program for their learners.

The current study does have some limitations. The sample size (*n* = 7), particularly the representation of female surgeons (*n* = 1) is limited and may not be representative of expert surgeons as a whole. Regarding our inclusion of just one female surgeon, this may reflect the lack of female representation in leadership positions in surgical societies nationally. In a recent retrospective analysis of trends in female leadership in national surgical societies from 2008 to 2018, it was reported that just 13.2% of leadership positions were held by women [26]. While we only included one female surgical expert in the current study, this surgeon represents 14.3% of the study sample, which aligns with this larger inequity of female representation in societal leadership positions. In regards to our small sample size in general, we only sampled senior-level surgeons in this study (i.e., at least 15 years of experience, post-training) and did not capture the use of mental skills by junior surgeons, which may not be representative of surgeons’ use of mental skills in general. However, most experts interviewed for this study described very similar processes of using mental skills preoperatively and intraoperatively, which indicates that most expert-level surgeons have likely learned some variation of these adaptive skills and are using them in at least some capacity. Our decision to sample only experienced surgeons was intentional, as we endeavored to determine whether expert surgeons use mental skills in practice. Surgeons with less than 15 years of experience may not have fully formed stress-coping strategies (i.e., adaptively or otherwise), so while studying more junior surgeons’ use of mental skills is an intriguing avenue for future study, it did not align with our study objective, which was to define expert surgeons’ use of mental skills in practice.

Moreover, despite this study’s small sample size, the participating surgeons were from a variety of institutions and are considered institutional, national, and international leaders in their disciplines and epitomize elite-level surgical performance. Since all interviewed surgeons reported using mental skills and the overwhelming majority of this study’s expert surgeons attribute some of their success during challenging clinical situations to their use of mental skills, the applicability of mental skills to optimizing performance in surgery appears very salient. Interestingly, the one surgeon who reported not actively implementing mental skills, indicated that psychological factors, such as remaining calm and in control, were critical to maintaining their performance during challenging clinical situations. Furthermore, this surgeon acknowledged that they experience an automatic process of “flipping the switch” at the scrub sink, which enables them to focus effectively on the upcoming case. It is apparent then that even for those expert surgeons who may not intentionally be using mental skills, they may be learning mental skills serendipitously and implementing them as habits for performance subconsciously. In the future, our team plans to verify this hypothesis by identifying and interviewing surgeons who do not use mental skills explicitly to determine what alternative techniques they use to mitigate the effects of stress and optimize surgical performance, or if they subconsciously use mental skills.

Another limitation of this study is that the interview guide was provided to participants prior to the interview, which could have theoretically biased them to the content of the interviews. However, the guide was provided prior to the interviews because mental skills are often used subconsciously, and participants may have had difficulty verbalizing the process of how they use mental skills. Having the interview guide ahead of time may have helped the surgeons consider their possible use of mental skills more profoundly thus leading to the collection of rich data.

## Conclusion

To perform and lead the team effectively, expert surgeons are regularly using mental skills preoperatively and intraoperative to mentally prepare for surgical cases, to manage the deleterious effects of over arousal, such as cognitive overload, to maintain working memory capacity and situation awareness on case-relevant stimuli, and to convey a calm demeanor to the surgical team. Although the participants in our study had no formal mental skills training during residency, the majority advocated for the inclusion of mental skills during surgical residency training.

## Appendix A. Interview Guide

A. General thoughts on mental skills

1. *Memorable Performance Moments*

*When you think of your most memorable moments performing as a surgeon, what comes to mind?*

- *Positive example*
- *Negative example*

2. *What do you consider to be the mental, psychological and emotional factors that influence successful surgery?*
3. *How did you learn the mental skills and techniques that you use?*

B. Ideal Performance State

In sports, performers often talk about their Ideal Performance State (IPS), which is their personal, unique combination of emotions and feelings when they perform their best.

1. *What is your Ideal Performance State for surgery?*
2. *Is your Ideal Performance State different for different procedures or phases of a surgical procedure?*
3. *Do you have any routines or techniques that you have developed to help achieve your Ideal Performance State?*

C. Dealing with stress

1. *Do you ever experience stress in the OR or related to surgery? In what ways can surgery be stressful?*
2. *Can you provide a specific example(s) of what becomes difficult when experiencing stress?*
3. *How do you manage stress in the OR?*
4. *Do you have any specific techniques that you do when things start to get tense?*
5. *Do you think the manner in which you handle crises in the midst of a procedure influences trainees and your OR team? If so, how?*
6. *In retrospect, are there things that you wish you had done differently in handling stressful situations?*

D. Attention and Thought Management

1. *Do you have any techniques that you use for handling negative thoughts or distractions?*

E. Imagery and Mental Rehearsal

1. *Do you ever use mental rehearsal or imagery in preparation for a procedure?*
2. *If so, what do you do?*

F. Refocusing Strategies

1. *Do you have any techniques or ways that you have developed to handle distractions or those unexpected events of a procedure or when things do not go as planned?*

G. Power of Routines

1. *How do you get ready for surgery?*
2. *Do you have any routines that you’ve developed to prepare for a procedure?*
3. *Do you have any routines that you use to help at transitions within a procedure (e.g., returning from a bathroom break; transitioning between laparoscopic and the Da Vinci machine; reassuming performance after another surgeon or resident has been performing the operation)*

H. Miscellaneous

1. *How important are mental skills and mental preparation for surgical success?*
2. *Do you have any specific mental techniques or strategies that you presently teach your trainees?*
3. *What advice would you offer surgical residents regarding mental skills and mental preparation?*
4. *Do you believe that trainees should have mental skills training? If so, when is the optimal time to provide mental skills training?*

## Declarations

### Data Availability

Data from this study will not be made publicly available to protect the privacy of study participants. Data will be made available to researchers upon reasonable request.
